# Developmental Differences in the Relationship Between Visual Attention Span and Chinese Reading Fluency

**DOI:** 10.3389/fpsyg.2019.02450

**Published:** 2019-11-06

**Authors:** Chen Huang, Maria Luisa Lorusso, Zheng Luo, Jing Zhao

**Affiliations:** ^1^Key Laboratory of Learning and Cognition, School of Psychology, Capital Normal University, Beijing, China; ^2^Unit of Neuropsychology of Developmental Disorders, Scientific Institute IRCCS E. Medea, Bosisio Parini, Italy

**Keywords:** Chinese reading development, visual attention span, silent/oral reading fluency, sentence reading, single-character reading

## Abstract

It has been suggested that there is a close relationship between visual attention span (VAS) and fluent reading. This relation may be modulated by participants’ age, and exhibits various patterns in different reading modes (i.e., oral vs. silent reading) and different reading levels (e.g., sentence vs. character/word levels). Moreover, the modulation effects from the above factors might be more remarkable in the framework of languages with a deep orthography. Therefore, the present study investigated the developmental pattern of the relationship between VAS skills and reading fluency in Chinese, a language with particularly deep orthography, by recruiting 292 participants from primary schools, middle schools, and universities. Two tests were utilized to assess fluent reading skills at the single-character and sentence levels with oral and silent reading modes. A visual 1-back task was adopted to reflect VAS capacity with non-verbal stimuli and no verbal response. Results showed that the VAS capacity of low-grade primary school students could significantly account for the variance in single-character reading fluency in the oral mode and that it was a significant predictor of sentence reading fluency in the oral mode among high-grade primary school students. VAS abilities of middle school students allowed a unique and stable prediction of their silent sentence reading. With increasing reading ability, VAS skills of adults showed significant and similar predictive power for estimating the variations in fluent sentence reading in both silent and oral modes. These results revealed developmental changes in the contribution of VAS to fluent reading in Chinese, and provided evidence unveiling whether the underlying mechanisms of oral and silent reading were shared or different.

## Introduction

Reading fluency refers to the ability to read rapidly and accurately to comprehend text (Klauda and Guthrie, 2008; Langer et al., 2013). However, some individuals cannot develop fluent reading skills, which can have severe academic, economic, and psychosocial consequences (Fraga González et al., 2015). Hence, it is necessary to explore the developmental mechanisms of reading fluency to help these struggling readers improve their skills in fluent reading. Fluent reading procedure involves simultaneous visual processing of several orthographic units (Lobier et al., 2012, 2013), which mainly reflects the capacity of the visual attention span (VAS, Bosse et al., 2007). The VAS refers to the amount of distinct visual elements that can be processed in parallel in a multi-element array (Bosse et al., 2007; Bosse and Valdois, 2009; Lobier et al., 2012, 2013; van den Boer et al., 2015). The connectionist multi-trace memory model of polysyllabic word reading proposed by Ans et al. (1998) provides a possible explanation for the relationship between VAS and reading fluency (especially for the word/lexical-level of fluent reading). According to this model, there are mainly two reading procedures (i.e., global and analytic reading modes). These two reading procedures differ in the size of the visual attention window from which orthographic information is extracted. In the global reading procedure, the window size of visual attention extends over the whole string, further contributing to the generation of the entire phonological output; by contrast, in the analytic reading procedure, the visual attention window narrows down to serially process single elements of the visual string, and to encode the relationship between orthographic and phonological sub-lexical segments (Ans et al., 1998; Bosse et al., 2007). The fluent reading procedure involves attention allocation across letters, and then VAS capacity limits the number of letters that can be processed in parallel during fluent reading, which in turn affects the processing of orthographic and phonological representations of the sequence.

Previous studies have typically adopted whole/partial report tasks and modified paradigms to measure VAS capacity, thus further examining the relationship between VAS and fluent reading. In the whole report paradigm, participants are required to report as many letters as possible from a previously presented five-letter string regardless of order; the relevant scores include the number of 5-letter strings accurately reported, as well as the number of letters accurately reported across all trials. In the partial report paradigm, the task is to report a single letter of a string that was presented at a cued position, and the number of letters accurately reported is regarded as the final score (Bosse et al., 2007). The higher the scores are, the better the visual attention span is. It has been reported that the performances of French children and Portuguese children aged 9–11 years in whole/partial report tasks were closely related to their oral word reading efficiency and text reading speed, revealing a close association between VAS skills and oral reading fluency at the word and text levels (French: Bosse et al., 2007; Lobier et al., 2013; Zoubrinetzky et al., 2014, 2016; Saksida et al., 2016; Portuguese: Germano et al., 2014). However, the traditional whole/partial report tasks use letters as target stimuli and adopt oral report as response, thus measuring aspects that belong to linguistic processing required by oral reading fluency (such as articulation or phonological processing) instead of relating to visual attention (Ziegler et al., 2010). Accordingly, some researchers have designed visual categorization and 1-back tasks on the basis of the partial report task by using symbols as non-verbal stimuli and with non-verbal responses to examine the pure processing of VAS. Participants were required to judge whether the target stimulus was in the previous symbol string (visual 1-back task), and/or to count the number of elements of a string belonging to a previously defined target category (the visual categorization task). The responses in these modified tasks (e.g., visual categorization task) were correlated with performance in traditional VAS tasks (Lobier et al., 2012). Relevant studies with the modified VAS tasks still found a significant correlation between VAS performance and word and text reading fluency among typically developing children in Arabic (Lallier et al., 2018), Spanish (Antzaka et al., 2017), French (Lobier et al., 2012), as well as among adults in Chinese (Zhao et al., 2017). Additionally, the findings of studies on individuals with reading fluency difficulties suggest a close relationship between VAS and reading fluency. Relevant research found that poor behavioral performance was reflected in lower accuracy, longer reaction times, and poor sensitivity of the VAS tasks in French, English, Portuguese, and Chinese children with reading fluency difficulties compared to their age-matched controls (Bosse et al., 2007; Germano et al., 2014; Zoubrinetzky et al., 2016; Zhao et al., 2018b). Furthermore, some researchers conducted a position-based analysis on relevant performance in each position of string stimuli in the VAS tasks to depict possible patterns of visual attention distribution and to explore its relation to reading performance. It has been found that, the inverted “V” shape of visual attention distribution, which means highest scores in the third position of the string and a decrease in performance with increasing eccentricity, is usually related to VAS tasks with non-verbal stimuli. By contrast, the “W” shape (i.e., the scores in the second and fourth positions lower than those in the other positions) is mainly related to VAS tasks with verbal stimuli such as digits and letters (Tydgat and Grainger, 2009; Ziegler et al., 2010). Furthermore, individuals with reading difficulties exhibited an atypical pattern of their visual attention distribution in VAS tasks as compared to that of the chronological age controls (Zhao et al., 2018b). The differences between the various stimuli and between groups with different reading abilities could reveal the possible modulation of reading experience on visual attention distribution as expressed by VAS. Additionally, a case study of a VAS intervention on a French girl with reading dysfluency reported that VAS-related training resulted in normalization of the pattern of her visual attention distribution in the VAS task, a faster identification of words and an improvement in text reading, which suggested a possible causal link between VAS and fluent reading performance to some extent (Valdois et al., 2014).

However, other studies did not replicate the finding of a strong link between VAS and fluent reading processes. By using traditional whole/partial report tasks and the visual 1-back task to measure VAS skills, it was found that 9-year-old German children and Arabic-, Spanish-, and Hebrew-speaking adults did not exhibit significant correlations between VAS and oral word/text reading speed (Awadh et al., 2016; Yeari et al., 2017; Banfi et al., 2018). Similarly, a non-significant group difference in VAS performance was found between dysfluent readers and non-impaired readers (Hawelka and Wimmer, 2008; Mi, 2016; Yeari et al., 2017; Banfi et al., 2018). Moreover, a recent longitudinal study found that the performance in whole/partial report tasks of 8-year-old Dutch children could not significantly predict their reading outcomes 1 year later, nor did reading fluency affect later VAS development (van den Boer and de Jong, 2018). The authors suggested that the absence of cross-lagged effects between VAS and reading performance thus indicated that the development of VAS and reading ability during this period were largely independent (van den Boer and de Jong, 2018). These findings challenged the relationship between VAS and fluent reading.

Since the orthographic depth of background language has been suggested to potentially modulate the relationship between VAS and reading (Awadh et al., 2016), the conflicting findings in the existing literature described above might be associated with differences in orthographic depth. Studies that found a significant relationship between VAS and fluent reading were usually in the context of languages with deep orthography (e.g., French, Chinese), while studies showing opposite results, that is, that there may be no relationship between VAS and reading fluency, were mostly in languages with shallow orthography (e.g., German, Spanish, and Hebrew). In languages with shallow orthographies, readers apply the rules of grapheme-to-phoneme correspondence (GPC), in which phonological regularity characterizes small orthographic units. By contrast, in languages with deep orthographies, larger orthographic units are more informative than smaller ones (Ziegler and Goswami, 2005). Accordingly, it could be said that VAS may contribute more to fluent reading in languages with deep orthographies. In shallow orthographies, other attentional processes involving visual attention orienting or focusing rather than VAS may be more crucial to reading, as shown by, e.g., studies on Italian dyslexic children (Facoetti et al., 2000, 2006). Several findings from cross-linguistic studies support the above inference to some extent. Lallier et al. (2016) found that French (deep orthography)-Basque (shallow orthography) bilingual children showed more efficient processing in tasks regarding VAS than Spanish (shallow orthography)-Basque (shallow orthography) bilingual children. Meanwhile, in the study of Awadh et al. (2016), Arabic, French, and Spanish adults (the orthographic depth of French being deeper than that of both Spanish and Arabic) were recruited, and the results showed that only the VAS skills of French adults were related to their scores on an oral reading fluency test. Moreover, the intervention study from Valdois et al. (2014) demonstrated that VAS training may primarily enhance the fast global-reading procedure, with a stronger contribution to reading processes in French compared to Spanish. The aforementioned findings support the idea that VAS may play a more notable role in fluent reading in languages with deep orthographies (Awadh et al., 2016; Lallier et al., 2016).

Additionally, age-related changes may modulate the relationship between VAS and fluent reading. A developmental study by Bosse and Valdois (2009) found that VAS exerted an increasing and sustained influence on fluent reading across grades in primary schools. The authors suggested that VAS skills might have a long-term effect on the acquisition of specific orthographic knowledge. Similar results were reported in the study of van den Boer et al. (2015), in which VAS was found to be a significant predictor of word reading fluency for the second and fifth graders of primary schools, and the role of VAS in word reading seemed to be more remarkable among advanced readers as compared to that of beginning readers. The above developmental studies revealed an increase with age in the relationship between VAS and reading fluency, which might have to do with the age-related changes in VAS skills and reading strategies. With increasing age, VAS capacity would gradually develop to a mature level (Bosse and Valdois, 2009). Meanwhile, as reading experience develops (especially for languages with deep orthographies), there is a transition from relying on the sub-lexical decoding strategy of letter-by-letter spelling to depending on a global lexical route (Frith, 1985). Larger visual attention span may contribute more to a global coarse-grain strategy than to a sub-lexical, small-grain strategy and play a more important role in higher grades (Bosse and Valdois, 2009). Since previous developmental studies usually recruited children (i.e., advanced/developing readers) as participants, their VAS skills and reading strategies were probably still developing. Therefore, whether mature VAS plays a sustained role in reading fluency even at the later stages of reading development in languages with deep orthographies is still an open question. To answer this question, future research will have to include adolescent and adult participants. Additionally, Mi (2016) found no significant differences in VAS between Chinese dyslexic children with reading fluency impairments and age-matched children. Given that the participants in their study were selected from the second to fifth grades in primary schools, covering a large age span, it is possible that the relationship between VAS and fluent reading in Chinese undergoes a developmental change within the period of primary school. Moreover, a longitudinal study (van den Boer and de Jong, 2018) found that there were significant correlations between VAS and reading fluency within each time point (i.e., T1: when participants were about 8 years old; T2: a year later when they were about 9/10 years old), but the VAS skills of 8-year-old children could not significantly predict their later reading fluency (van den Boer and de Jong, 2018). The non-significant prediction might be with the result of obvious developmental changes in VAS skills and reading strategies from 8 to 9/10 years of age. For example, the VAS of 8-year-olds may be still developing and rather limited, while their reading fluency skills could reach the stage relying on the globally lexical route at 9/10 years of age; in such case, the small VAS of 8-year-olds would not be a significant predictor for later reading performance using the lexical route. The above findings also suggested that we should pay more attention to the early stages of development. We could divide the children into beginning and developing readers by the age of about 9/10 years, so as to further and fully explore the relationship between VAS and reading fluency at the early stages of reading development.

Another aspect that should be carefully considered is the influence from different reading levels and reading modes on the developmental relationship between VAS and reading fluency. Fluent reading occurs at various levels, including lexical and sentence/passage levels (Kim et al., 2011). Lexical-level fluency (or list reading fluency) can be measured by having the individual read lists of words/characters as quickly and accurately as possible, reflecting fluency at decoding words/characters in isolation (Kim et al., 2011). Reading fluency at the sentence/passage level mainly concerns word identification and decoding in context. Moreover, there are primarily two modes of reading fluency: oral reading fluency and silent reading fluency (van den Boer et al., 2014). During oral reading, individuals (especially beginning or developing readers) aim to pronounce every word correctly, while the comprehension of text is a secondary goal, so this process focuses more on orthographic-to-phonological mapping; whereas, the main goal in silent reading is to comprehend and assimilate the meaning of the text, a process relying more on grapheme-to-semantic decoding (Galin et al., 1992; Snellings et al., 2009; van den Boer et al., 2014). As the reading experience develops, the main level upon which reading fluency is based becomes increasingly complex; the prominent reading mode gradually shifts from oral mode to silent mode (Kim et al., 2011; van den Boer et al., 2015). In details, as indicated by the Chinese teaching programs in primary and middle schools (Wang and Li, 1999; Wang, 2007), low graders are taught to use the oral mode in their daily reading and to accurately and fluently read aloud in an item-by-item order with expression to support the comprehension of text with simple construction. From the end of the second grade of primary school, the students start to silently read simple texts, but the main reading mode is still the oral mode, until the end of primary school. Middle school students are required to combine the oral and silent reading modes. However, the oral reading procedure is still frequently used in daily reading by middle school students to support correct understanding of the general meaning of more complex texts, in which phonological retrieval still occurs in a word-by-word/character-by-character method. With continued practice, middle school students develop greater silent reading skills. As to adult readers, they tend to be proficient in both the oral and the silent reading modes. The oral and silent reading of simple and complex materials at different developmental stages might require different window sizes of the visual attention span. For example, character-by-character oral reading in early developmental stages may correspond to a small VAS window, while the silent reading of multiple orthographic units in parallel by skilled readers may require a wide VAS window.

Considering that participants’ age as well as reading levels/modes would exert an influence on the relationship between VAS and reading fluency, and that the modulations from the above factors have been suggested to be more notable in languages with deep orthographies, the present study investigated the developmental trajectory of the relationship between VAS and reading fluency in Chinese among beginning readers, developing readers, and skilled readers. In contrast to alphabetic languages, Chinese, as a logographic writing system, lacks strict GPC rules and has a particularly deep orthography. Chinese characters are visually complex and allow creation of sentences without inter-word spacing (Liu et al., 2015, 2016). It has been suggested that effective attention is required to process detailed visual information and identify characters with ambiguous word boundaries in a sentence, which is particularly important for reading fluency in Chinese, as it contributes to the efficient mapping between orthography and phonology/semantics. Thus, it is reasonable to assume that VAS may play an important role in Chinese reading fluency. Moreover, as reading in Chinese develops, the direct connections between the visual forms and the corresponding meanings of Chinese characters become well established, which increases the involvement of morphological processing (Huang and Hanley, 1995; Siok and Fletcher, 2001). As VAS has been suggested to exert a more significant influence on global, visual-semantic mapping (Zhao et al., 2018a), it is interesting to explore whether its contribution is more crucial for Chinese reading fluency in skilled readers than in the early stages of reading development. In the present study, a visual 1-back task with non-verbal figure stimuli and non-verbal response was adopted to assess VAS skills. Reading fluency in both the oral and silent modes was systematically tested at the single-character and sentence levels. Referring to relevant research using VAS tasks with symbol/figure stimuli (Ziegler et al., 2010; Zhao et al., 2018b), we predicted that the distribution pattern of VAS might gradually develop to a typically inverted “V” shape with age. Moreover, in line with previous literature (Lallier et al., 2013; van den Boer et al., 2014, 2015; Awadh et al., 2016; Zhao et al., 2017, 2018b; van den Boer and de Jong, 2018), it could be expected that VAS ability exhibits a special relationship with Chinese oral reading fluency at single-character level in the early developmental stages. With increasing age, VAS skills might be more markedly related to Chinese sentence reading fluency and this prediction would be more significant for the silent reading mode as compared to the oral reading mode; moreover, we predicted that this relationship would be more evident for readers in later developmental stages.

## Materials and Methods

### Participants

Four groups of participants were recruited in the present study: 85 children (45 boys) from the second and third grades of primary school as beginning readers (younger than 9 years old), 78 children (37 boys) from fourth to sixth grades of primary school as developing readers (9–11 years old), 66 middle school students as adolescent skilled readers (34 boys), and 63 undergraduates as adult skilled readers (26 males). All participants were right-handed and had normal or corrected-to-normal vision without ophthalmologic or neurological abnormalities. Detailed information on each group is presented in Table 1. Informed consent was obtained for the primary and middle school students from their parents and teachers before assessment took place. The research project was approved by the Research Ethics Committee of the School of Psychology, Capital Normal University. The study was carried out in accordance with the relevant guidelines and regulations.

**Table 1:** Descriptive statistics of each condition.

Characters	(1) Lower graders *N* = 82	(2) Higher graders *N* = 77	(3) Middle school students *N* = 65	(4) Undergraduates *N* = 61	*F* values	Groups comparison
	Mean (SD)	Mean (SD)	Mean (SD)	Mean (SD)		
**Age** (years)	8.47(0.85)	11.2(0.99)	14.31(0.68)	23.23(1.94)	2709.34^***^	(1) < (2) < (3) < (4)
***Single-character level***							
Oral speed (c/min)	93.51(18.53)	113.14 (22.47)	129.58 (19.11)	138.96 (32.02)	50.72^***^	(1) < (2) < (3) = (4)
Silent speed (c/min)	139.65 (41.87)	165.81 (65.50)	184.35 (55.30)	214.75 (72.66)	17.96^***^	(1) < (2) = (3) < (4)
***Sentence level***						
Oral speed (c/min)	152.64 (29.51)	189.24 (64.09)	251.28 (77.66)	304.04 (92.75)	62.73^***^	(1) < (2) < (3) < (4)
Oral accuracy	0.80 (0.07)	0.84 (0.07)	0.87 (0.06)	0.92 (0.03)	43.53^***^	(1) < (2) < (3) < (4)
Silent speed (c/min)	264.91 (90.59)	352.67 (156.41)	473.93 (217.40)	507.99 (205.11)	29.23^***^	(1) < (2) < (3) = (4)
Silent accuracy	0.81 (0.05)	0.83 (0.08)	0.87 (0.06)	0.92 (0.04)	39.93^***^	(1) = (2) < (3) < (4)
**VAS tasks**						
VAS RT(ms)	1,698 (436)	1,360 (330)	1,051 (209)	944 (265)	64.03^***^	(1) < (2) < (3) = (4)
VAS *d*-prime	0.53 (0.36)	0.55 (0.39)	0.57 (0.40)	0.68 (0.30)	2.06	—
**RAN (s)**	21.66 (3.33)	10.52 (2.39)	8.80 (1.28)	8.04 (2.42)	462.47^***^	(1) < (2) < (3) = (4)
**Reasoning ability**	RSPM	RSPM	Math test score	Math test score		
	34.76(11.75)	41.17(9.36)	67.24(17.69)	108.85(10.60)	—	—
**Language level**	Chinese character test	Chinese character test	Chinese test score	Chinese test score		
	1130.72(433.56)	2647.05(593.52)	68.97(9.07)	112.56(6.47)	—	—

*The descriptive statistics for the valid participants who responded above the chance level in the visual 1-back task. Measure units are in the parentheses for each item in the “Characters” column. RAN, the scores in the digital rapid automatized naming test. Reasoning ability, the performances of RSPM in children from primary schools, scores in the latest final math examination for middle school students, and the math scores of college entrance examination for university students. Language level, the scores in Chinese character recognition test in primary school students, the scores in the latest final Chinese examination for middle school students, and the scores of college entrance Chinese examination for university students. VAS RT, correct reaction times in the visual 1-back task. VAS *d*-prime, *d*′ values in the visual 1-back task*.

*** *p < 0.001*.

### Reading Fluency Test

The present study was designed to measure fluent reading skills at the single-character and sentence levels. Particularly, according to relevant literature (Salasoo, 1986; Zhao et al., 2017), a character-list reading task and a sentence verification task were used to test reading fluency at the single-character and sentence levels, respectively. Moreover, to further explore the underlying mechanisms of oral and silent reading, fluent reading tests at the single-character and sentence levels were conducted in both the oral and the silent reading mode. Participants performed the same tasks in both the oral and silent reading conditions to control for the effects of different reading materials. The methods employed to reduce possible practice effects using the same materials are stated in the “Procedure” section.

#### Single-Character Level

The reading fluency test at the single-character level consisted of a character-list reading task in both oral and silent modes with a time limit of 1 min, as described in Zhao et al. (2017). The split-half reliability was 0.93. Four-hundred Chinese characters intermixed with 13 non-characters in random order were presented on a white sheet; the non-characters were introduced to ensure validity of the silent reading task. The main task for the participants was to silently read the Chinese characters as accurately and quickly as possible within the time limit while crossing out the non-characters when they were encountered. At the end of the task period, participants were required to mark the last item they had read. The final score for the single-character reading fluency test was the number of items they had read minus the number of errors, which included non-characters that were not correctly identified as well as real characters that were incorrectly crossed out. The score was expressed as the number of characters correctly read in 1 min (c/min).

#### Sentence Level

A sentence verification task was utilized to measure reading fluency at the sentence level (Zhao et al., 2017, 2018b). The split-half reliability was 0.85. There were four sentences in the practice session and 50 sentences in the formal experiment. Similar to other reading fluency tests in Chinese (e.g., Xue et al., 2013), sentence length was arranged from short to long across the test, and the number of characters in each sentence varied from seven to 22 in the present study. The task included obviously true or false sentences. Half of the sentences were true. All of the characters were high-frequency ones. The grammatical structure of all the sentences was “subject+verb+object[+prepositional phrase in some of the sentences],” such as “池塘在冬日里开满了荷花,” meaning “*The pond is full of lotuses in winter*” (this sentence is false), or “一年 有十二个月,” meaning “*There are twelve months in a year*” (this sentence is true). Sentence reading fluency had been examined through a paper-and-pencil test in previous studies (e.g., Kim et al., 2011; Pan et al., 2011, 2016; Xue et al., 2013). Participants were required to silently read a list of sentences within a time limit (e.g., 3 min); after reading each sentence, participants were asked to judge the correctness of this sentence. The final scores of the test were the number of the correctly marked sentences, or the total number of characters in the correctly marked sentences within the time limit. However, the tasks to be performed within this time limit included not only fluent reading but also sentence judgment. Therefore, a computerized test of reading fluency was used, programmed with E-prime software 1.0 (Zhao et al., 2017), in which assessment of reading fluency and correctness judgment were separated from each other. Accordingly, the relevant indexes of this reading fluency test included speed of fluent reading, as well as judgment accuracy just like previous studies. The testing procedure for the sentence reading test was as follows: a 500-ms fixation cross was displayed at the center of the screen, followed by the appearance of a complete, single-line-long sentence. Participants were asked to read the sentence aloud or silently as accurately and quickly as possible and to press the space bar once they had finished reading the sentence. Reading time was recorded as the interval between the beginning of the sentence presentation and the time when the space bar was pressed; the two time points (i.e., the beginning of the sentence presentation, and the time of pressing the space bar) could be automatically recorded by E-prime software. We could then compute sentence reading speed as the ratio between the number of characters in one sentence and the corresponding reading time. Consistent with the unit of single-character reading speed and referring to relevant literature on sentence/text reading (e.g., Kim et al., 2011; Kim and Wagner, 2015), the reading speed unit was converted into the number of characters read in 1 min (c/min). For example, a participant orally reads one sentence consisting of 20 characters, and the reading time is recorded as 6 s (i.e., 0.1 min). Accordingly, the participant’s reading speed is 20 characters/0.1 min =200 c/min. Following the pressing of the space bar, participants were instructed to judge the veracity of the sentence by pressing the “f” key if it was true and the “j” key if it was false. The accuracy of the veracity judgment was also recorded. This test was presented by means of a Dell laptop, and the average reading speed and judgment accuracy were recorded for further analysis.

### Visual Attention Span Test

**Figure 1:**
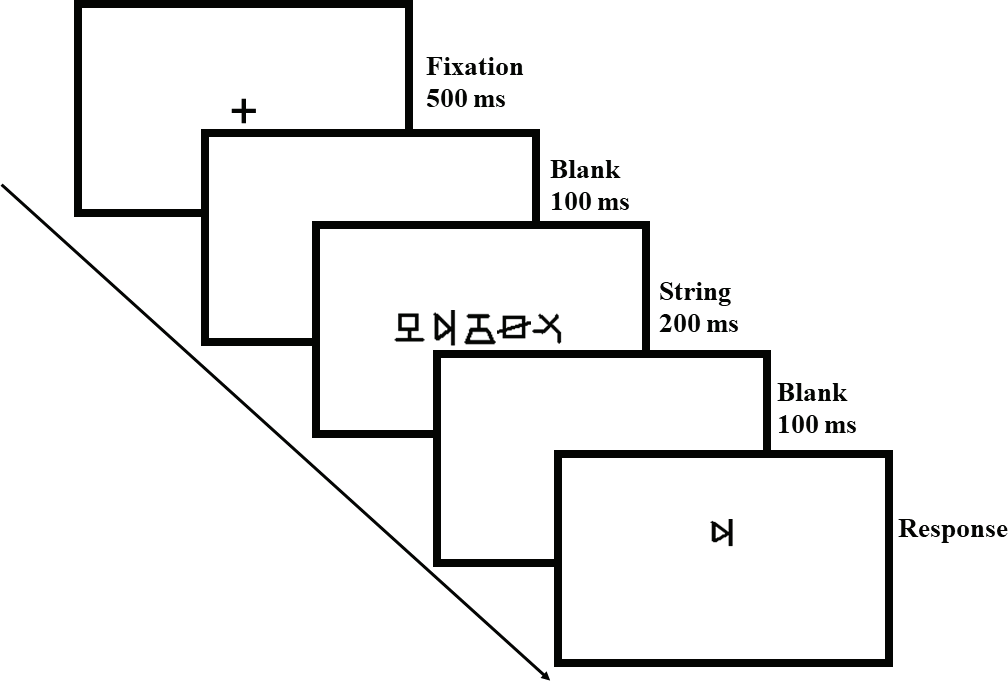
The presentation format of each trial in the visual 1-blank task. In each trail, a 500-ms fixation was first presented in the screen center, and then there was a 100-ms blank, which was followed by a probe of the five-figure string centering on the screen for 200 ms. The string was replaced by a 100-ms blank, and finally, the target of a single figure appeared below or above (half of the trails) the median horizontal line. Participants were asked to press different keys to judge whether the target figure was present in the above string or not.

We used a visual 1-back task (Zhao et al., 2017) to measure VAS skills with 15 figures as non-verbal stimuli (Table 2). The test-retest reliability was 0.81. The visual complexity of the 15 figures was evaluated by 20 undergraduates (12 female) who did not take part in the formal experiment. The result of the evaluation, which used a 6-point rating scale (1 = the figure is extremely simple, 6 = the figure is extremely complex), showed the visual complexity to be 2.27 ± 0.05 on average, revealing low and middle degrees of complexity. The visual complexity was not significantly different between any two of the 15 figures [*F*(14, 266) = 0.48, *p* = 0.81, *η*^2^ = 0.05]. The complexity rating score of each figure is shown in Table 2. A list of 80 five-figure strings was created randomly using the 15 figures, and no figures in one string were the same. The stimuli were presented in black on a white background with E-prime 1.1 software on a Dell laptop. The display resolution was set to 1,024 pixels × 789 pixels with a monitor refresh rate of 75 Hz. The strings subtended 7.9° × 0.8° at a viewing distance of 50 cm, with a center-to-center distance between each adjacent figure of 1.7°. The presentation format of each trial was consistent with the study of Zhao et al. (2017, 2018b). In each trial (see Figure 1), a fixation cross was first presented at the center of the screen for 500 ms; 100-ms blank premask then appeared, followed by the five-figure string presented at the center of the screen for 200 ms and then another 100-ms blank postmask; finally, a single figure was presented below or above (half of the trials each) the median horizontal line. Participants were required to press “z” as quickly and accurately as possible if the single figure was present in the previous string and “b” if it was not. After pressing the key, a blank screen appeared for a random interval (between 1,000 to 1,500 ms). There were 10 practice trials; the formal test consisted of 80 randomly presented trials, consisting of 50 target-present trials and 30 target-absent trials. The means of the response time and accuracy in each position of a string were recorded. Moreover, we further computed *d*-prime values based on accuracy for further position-based analysis. The procedure of computing relevant *d*′ values was as follows: (1) calculating the hit rate and false alarm rate. The hit rate in one position condition is just the corresponding accuracy in this condition, and the false alarm rate was the difference between 1 and correct rejection rate (i.e., the accuracy of correct response when the target was absent); (2) converting the rate values to z scores through the standard normal distribution function, thus obtaining *Z*_hit_ and *Z*_false alarm_ scores; (3) computing *d*-prime values. *D*′ values in one position condition equals the difference between relevant *Z*_hit_ and *Z*_false alarm_.

**Table 2:** Rating scores of visual complexity for each of the 15 figures.

No. Figure	F01	F02	F03	F04	F05	F06	F07	F08	F09	F10	F11	F12	F13	F14	F15
Figure	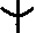	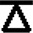	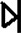	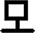	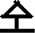	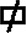	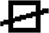	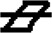	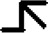	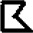	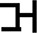	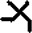	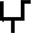	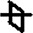	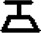
Visual complexity	2.10 ±0.28	2.20 ±0.20	2.00 ±0.26	2.30 ±0.21	2.40 ±0.27	2.00 ±0.37	2.10 ±0.23	2.20 ±0.25	2.20 ±0.39	2.30 ±0.37	2.40 ±0.31	2.30 ±0.34	2.40 ±0.27	2.60 ±0.31	2.60 ±0.31

### Measurement of Control Variables

General cognitive abilities predict reading development (e.g., Steinbrink et al., 2014) and might also be involved in the execution of visual spatial attention tasks. Moreover, individual differences in general language ability may affect the relationship between visual attention span and reading fluency. Thus, to disentangle the unique contribution of VAS to reading fluency from that of more general factors, reasoning ability (as a measure of general cognitive ability) and language processing level were assessed and used as control variables when analyzing the data. Reasoning ability was measured with the Raven’s Standard Progressive Matrices (RSPM) in the primary school children, and the scores in the latest final math examination were used as measures of general cognitive ability for middle school students; finally, we regarded the math scores of their college entrance examinations of the university students as measures of their general cognitive ability. A standardized measurement of a Chinese character recognition test was used to estimate vocabulary size in the primary school children, which according to previous studies, could reflect their language processing level (e.g., Spencer et al., 2019). The scores in the latest final Chinese examination were used as an index of the relevant language ability for the middle school students, while the scores of the college entrance Chinese examination were used as an estimation of the language processing levels for the university students. Moreover, rapid naming as another important predictor of fluent reading was considered and controlled (Liao et al., 2008; van den Boer et al., 2014). It has been suggested that traditional report tasks in VAS involve articulation, phonological processing, and visual-verbal transfer, similar to the cognitive processes involved in rapid naming (Moll et al., 2009; Ziegler et al., 2010; van den Boer et al., 2014; Nielsen and Juul, 2016; Chen et al., 2019), although these two cognitive skills differ from each other in some critical aspects (see Bosse and Valdois, 2009 for more details). The current study used a visual 1-back task with figures as non-verbal stimuli and key pressing as non-verbal response, which show less overlap with processes involved in rapid naming procedure as compared to traditional VAS tasks. Nevertheless, rapid naming and the visual 1-back task still seem to share some processes, such as quick visual identification of the stimuli (Powell et al., 2007; Bosse and Valdois, 2009; van den Boer et al., 2015). Therefore, the present study also examined rapid naming performance and regarded the relevant score as one of the main control variables so as to better explore the independent contribution of VAS to reading fluency. Detailed descriptions of these tests follow.

#### Rapid Automatized Naming Task

Based on previous research (Salasoo, 1986; Liao et al., 2008; Franceschini et al., 2012), a digit RAN task was utilized. Two digital matrices were generated containing the Arabic digits 2, 4, 6, 7, 9. Each matrix consisted of six rows, with the five digits presented in a random order in each row. The digits were typed in 36-point, Times New Roman font on an A4 sheet. Participants were asked to read each of the digits as quickly and accurately as possible from left to right, row by row. Reading time was recorded by a trained tester using a stopwatch, and average completion time was computed as the final score of the test.

#### Reasoning Ability Tasks

In the current study, we used different measures of reasoning for different age groups. Raven’s Standard Progressive Matrices (RSPM, Zhang and Wang, 1985) was used to measure the reasoning ability of low and high graders of primary school. The RSPM is a standardized test of non-verbal intelligence comprising five sets with 12 items in each set. Each item consists of a target matrix pattern with one missing element. Participants are required to select from six to eight alternatives to complete the patterns. The difficulty of the matrices increases as the test progresses. The raw score was the number of correct choices, which was used in the following data analysis. The math test for the middle school students required calculating the area and edge length of a triangle and a parallelogram and solving problems about linear functions and inequality. The maximum total score for the math test was 100, and each student was awarded a score by her/his own math teacher. The college entrance math examination was administered to adult participants and contained problems regarding solid geometry, analytic geometry, reasoning on number sequences, probability estimation, trigonometric functions, etc. The maximum score for the college entrance math examination was 150, which was marked by math teachers through a blind reviewing procedure. Thus the math examinations for the middle school students and adults mainly required problem-solving, reasoning, and operational capabilities, which to some extent could be regarded as indicators of non-verbal intelligence.

#### General Language Ability Tasks

Different measures indexing general language ability were used across age groups. Consistent with relevant literature (Spencer et al., 2019), the Chinese Character Recognition Test (Wang and Tao, 1996) was adopted to measure general language ability in the primary school students. In this paper-and-pencil test, participants were required to write a compound word based on a target morpheme on a sheet of paper. There were 10 groups of characters in this test, each corresponding to a different reading difficulty level. Each correct response was given one point. The sub-score for each group was calculated by multiplying the total points by the corresponding difficulty coefficient. The final score was the sum of the sub-scores of the 10 groups. The Chinese character test for the middle school students consisted of questions about phonetic notation, synonyms, vocabulary, text comprehension, and writing. The maximum score of the Chinese test for the middle school students was 100, and each student was awarded a score by her/his own Chinese teacher. The college entrance Chinese examination for the adults included comprehension of various types of passages, lexical manipulation and usage, and writing. The maximum score for the college entrance Chinese examination was 150, which was marked by Chinese teachers through a blind reviewing procedure. The tests for the middle school and undergraduate students involved vocabulary size, morphonology-based knowledge and writing skills, which were considered as measures of the general language processing levels of the participants.

### Procedure

Participants were tested individually in a quiet room. Oral and silent reading tasks for each level were separated by the VAS test (interval: approximately 15–20 min) to reduce possible practice effects in utilizing the same materials for the two reading modes. Therefore, there were four sessions in the present study: character reading fluency and sentence reading fluency tasks (oral/silent); the VAS task; character reading fluency and sentence reading fluency tasks (silent/oral); and the digit RAN task. The reading modes between the two sessions were reversed. For example, if we measured character reading fluency in oral mode and sentence reading fluency in the silent mode in the first session, then silent character reading and oral sentence reading were examined in the third session. Additionally, the test order of the RAN task was randomly arranged during the procedure. There was a 1-min rest between successive sessions. The total testing time was approximately 45–50 min for the primary school students, 40–45 min for the middle school students, and 30–40 min for the adult participants.

### Data Analysis

First of all, the datasets of seven participants (three low graders of primary school, one high grader of primary school, one middle school student, and two undergraduates) were discarded because they responded at or under chance level in the visual 1-back task. In particular, this chance level was computed though the formula of “δ*ln(*π*/(1 − *π*))” referring to relevant literature (Lee and Danileiko, 2014), where *π* is the true probability and *δ* represents expertise ranging from 0 to 1 contributing to reducing the probability toward 0.5. In the present study, *π* = 0.625, and we could not define the exact value for *δ*, so we only could compute and infer the range of the chance level to be changing from 0.5 to about 0.511 [i.e., the possible maximum for this chance level, which is computed in the condition of *δ* = 1, and 1*ln(0.625/(1 − 0.625)) ≈ 0.511]. We used a more strict value of 0.511 as the chance level to identify participants to be excluded because of invalid performance. That is, if one participant’s mean accuracy in the visual 1-back task was lower than 0.511, then his/her dataset would be excluded. Consequently, the datasets of the seven participants mentioned above were removed, while the data of the remaining participants were put into further pre-processing and analyses. As to the reaction time in the visual 1-back task, we firstly checked and discarded the correct reaction times shorter than 200 ms in order to exclude anticipatory responses (24 values excluded, about 0.17%); and then the absolute values of correct reaction times longer than 3 standard deviations above the average were excluded (97 values discarded, approximately 0.71%). We used *d*′ values instead of the accuracy in the following analysis because *d*′ values and accuracy were overlapping with each other to a great extent; meanwhile *d*′ values were a bias-free estimate of task sensitivity (Pammer et al., 2005; Lallier et al., 2016). The *d*-prime values and remaining reaction times at each position of the five-figure strings were regarded as indexes of VAS skills and were analyzed by two-way ANOVA—using position within a string (1–5) as the within-subject factor, group (low and high grade primary school students, middle school students, and undergraduates) as the between-subject factor—to compare the relevant VAS capacity across age groups and target positions. *Post hoc* analyses and multi-group comparisons were performed with Bonferroni correction. Moreover, in order to check how general reasoning ability affected ANOVA results on reaction times and *d*′ values of the visual 1-back task, we further conducted ANCOVAs separately within each age group with the raw scores of general reasoning ability as covariate. The results are reported in the Supplementary Material.

Reading fluency was measured at the single-character and sentence levels in both the oral and silent modes. The relevant index for the single-character reading was the speed of single-character reading. The measurements for sentence reading fluency included judgment accuracy in the sentence verification task, as well as the reading speed for each sentence. Specifically, the reading speeds for one sentence higher than 3 standard deviations from the average or lower than −3 standard deviations from the average were discarded (84 values excluded, about 0.69%), and then the remaining sentence reading speeds were averaged for further analyses of partial correlation and regression. Partial correlation analyses were conducted as preliminary analyses to examine the possible relationships between mean reaction times and *d*′ values in the VAS task and the reading skills within each age group, while controlling for participants’ age, general language ability, reasoning ability, and rapid naming speed. Finally, according to the significant results in the correlation analyses, regression analyses were conducted for each group to examine the ability of the average VAS measurements to independently predict fluent reading performance.

## Results

### Development of Visual Attention Span Skills

#### Reaction Times

**Figure 2:**
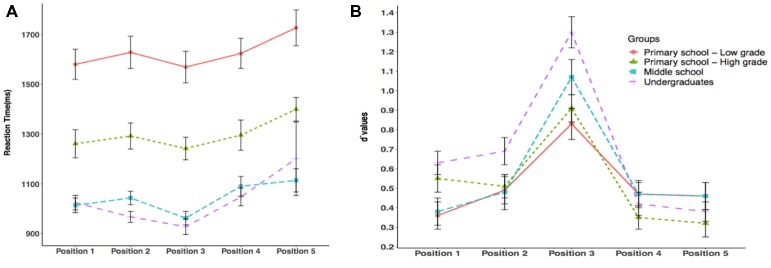
Position-based analyses on correct reaction times **(A)** and *d*′ values **(B)** of visual 1-back task for the four age groups. Standard error bars, 1 SE. Red solid line, lower graders from primary schools; green dot line, higher graders from primary schools; cyan dashed line, middle school students; purple dashed line, university students.

The results of ANOVA on the reaction times for each target position in the visual 1-back task (Figure 2A) showed a significant main effect of age [*F*(3, 279) = 43.25, *p* < 0.001, *η*^2^ = 0.31]. *Post hoc* analysis revealed that the middle school students and university students responded faster than the primary school students in both grade groups (*p’*s < 0.001) and that high graders of primary school showed shorter reaction times than low graders (*p* < 0.001); however, there was no significant difference between middle school students and adults (*p* = 1.00). The main effect of position was also significant [*F*(4, 1,137) = 10.19, *p* < 0.001, *η*^2^ = 0.03]. Planned multiple comparisons showed that the reaction time in the third position was shorter than that in the fourth and fifth positions (*p’*s < 0.001); participants also responded faster when the target was presented in the first and second positions compared to the fifth position (*p’*s < 0.05). There were no other significant differences in reaction times between positions (*p’*s > 0.1). The group × position interaction was not significant [*F*(12, 1,137) = 0.51, *p* = 0.91, *η*^2^ = 0.01].

#### *D*-Prime Values

The results of the two-way repeated measures ANOVA on the *d*′ values of the VAS task (Figure 2B) did not reveal any main effect of age [*F*(3,215) = 1.92, *p* = 0.13, *η*^2^ = 0.03]. The main effect of position was significant [*F*(4,877) = 63.48, *p* < 0.001, *η*^2^ = 0.22], and Bonferroni-corrected multiple comparisons showed that the *d*′ value of the third position of the string was higher than that of the other four positions (*p’*s < 0.001) and that the *d*′ value of the second position was higher than that of the fourth position (*p =* 0.03). Moreover, a significant interaction between target position and grade was found for the *d*′ value [*F*(12,877) = 2.70, *p* = 0.002, *η*^2^ = 0.04]. Simple effect analysis showed that the group difference was significant for the first [*F*(3,215) = 2.88, *p* = 0.04, *η*^2^ = 0.04] and third [*F*(3,215) = 6.11, *p < 0*.001, *η*^2^ = 0.08] positions. Multiple comparisons across groups of the *d*′ value in the first position showed that low graders from primary school exhibited lower *d*′ values compared to undergraduates (*p* = 0.04) without any other significant group differences (*p’*s > 0.1). These results also showed that undergraduates exhibited better performance in the *d*′ value of the third position than children from primary school (low graders: *p* < 0.001; high graders: *p* = 0.003) and that there was no other significant difference between groups for this *d*′ value (*p’*s *> 0*.1). Moreover, the position effect was significant for each age group. *Post hoc* analysis showed that primary and middle school students exhibited similar patterns in the position effect, that is, the *d*-prime scores of the third position were higher than those of the other four positions (*p’*s < 0.05), and there were no other significant differences across positions (*p’*s > 0.1). However, the undergraduate group, in addition to having their highest *d*′ value in the third position, also had significantly higher *d*-prime scores at the first and second positions than at the fourth and fifth positions (*p’*s < 0.05). To further examine whether the *d*-prime values were distinct from zero, one-sample *t*-tests were conducted within each position, condition, and each age group. The results showed that the mean *d*′ values at all levels were significantly above zero (*p’*s < 0.001).

### Regression Analyses on the Predictive Power of Visual Attention Span for Chinese Reading Fluency

In preliminary analyses, partial correlations were computed. Tables 3, 4 show partial correlation coefficients among all measures in each age group while controlling for participants’ age, general language ability, reasoning ability, and rapid naming speed. Of particular interests are the significant correlations between VAS skills and reading fluency in each condition. As shown in Tables 3, 4, VAS measures were significantly correlated with oral and silent reading fluency in both single-character and sentence levels among low graders of primary schools. The VAS capacity showed to be related to sentence reading fluency in either oral or silent mode for higher age groups. Concerning significant correlations between VAS and reading fluency, further analyses were carried out, namely, hierarchical regression analyses to examine the independent contributions of VAS skills to reading fluency in each group. In correlation and regression analyses, the transformed-rank data of reasoning ability and general language level were used because their originally continuous data were different across groups.

**Table 3:** Partial correlation among all measures in low and high graders of primary schools.

Characters	1	2	3	4	5	6	7	8
1. VAS_rt	—	0.16	−0.12	−0.07	−0.38^**^	−0.02	−0.23	0.06
2. VAS_d′	0.29	—	−0.04	0.03	−0.10	0.14	0.07	0.25^*^
3. O_char	−0.37^*^	−0.02	—	0.17	0.10	−0.03	0.04	0.17
4. S_char	−0.24	−0.39^*^	0.27	—	0.11	−0.10	0.13	0.12
5. O_senspe	−0.42^*^	−0.21	0.35^*^	0.19	—	0.14	−0.13	0.18
6. O_senacc	−0.38^*^	0.08	−0.41^*^	0.10	−0.24	—	0.19	0.67^***^
7. S_senspe	−0.29	−0.19	0.30	−0.04	0.28	−0.09	—	0.20
8. S_senacc	−0.36^*^	0.24	−0.33	−0.10	−0.01	0.47^*^	−0.29	—

*This partial correlation controls for age, general language ability, reasoning ability, and rapid naming speed. The correlation coefficients below the diagonal line represent data of lower graders, and the correlation coefficients above the diagonal line represent data of higher graders. VAS_rt, correct reaction times in the visual 1-back task; VAS_d′, *d*-prime values in the visual 1-back task; RAN, mean time recorded in the rapid naming test; O_char, oral character reading speed; S_char, silent character reading speed; O_sen, oral sentence reading speed; S_sen, silent reading speed*.

* *p < 0.05*;

** *p < 0.01*;

*** *p < 0.001*.

**Table 4:** Partial correlation among all measures in middle school students and undergraduates.

Characters	1	2	3	4	5	6	7	8
1. VAS_rt	—	0.08	−0.05	−0.08	−0.33^*^	0.04	−0.39^*^	0.14
2. VAS_d′	0.37^**^	—	−0.17	−0.19	0.17	−0.02	−0.07	0.03
3. O_char	−0.04	0.12	—	0.33^*^	0.32^*^	−0.06	−0.21	−0.06
4. S_char	0.02	−0.16	0.10	—	0.08	0.07	0.04	−0.08
5. O_senspe	−0.6	0.10	0.19	0.08	—	−0.12	0.03	−0.01
6. O_senacc	0.14	−0.04	−0.14	0.16	−0.12	—	−0.10	0.46^**^
7. S_senspe	−0.31^*^	0.04	0.11	−0.01	0.11	0.10	—	−0.02
8. S_senacc	0.12	0.10	−0.15	0.12	0.10	0.64^***^	0.10	—

This partial correlation controls for age, general language ability, reasoning ability, and rapid naming speed. The correlation coefficients below the diagonal line represent data of lower graders, and the correlation coefficients above the diagonal line represent data of higher graders. VAS_rt, correct reaction times in the visual 1-back task; VAS_d′, *d*-prime values in the visual 1-back task; RAN, mean time recorded in the rapid naming test; O_char, oral character reading speed; S_char, silent character reading speed; O_sen, oral sentence reading speed; S_sen, silent reading speed.

* *p < 0.05*;

** *p < 0.01*;

*** *p < 0.001*.

Table 5 displays the procedure for inputting the variables into the regression model. For the low graders of primary schools, most of the significant correlations did not survive in the subsequent regression analyses; an exception was reaction time in the visual 1-back task which was a significant predictor of unique variance of oral reading speed at the single-character level (5%). As to the high graders of primary school, VAS skill (i.e., the reaction times in the VAS test) significantly predicted the variance of oral sentence reading speed (11%). Reaction times by middle school students on the VAS test accounted for approximately 7% of their unique variance in silent sentence reading speed. The reaction times on the VAS test of the undergraduates accounted for 12% of the variance of silent sentence reading speed and 14% of the variance of oral sentence reading speed.

**Table 5:** Hierarchical regression analyses that estimated the predictive power of VAS on reading fluency at each age group.

Step and variable	Single-character level									Sentence level															
	Oral				Silent					Oral_speed				Oral_accuracy				Silent_speed				Silent _accuracy			
	*R*^2^	∆*R*^2^	∆*F*	*β*	R^2^	∆R^2^	∆F	*β*		*R*^2^	∆*R*^2^	∆*F*	*β*	*R*^2^	∆*R*^2^	∆*F*	*β*	*R*^2^	∆*R*^2^	∆*F*	*β*	*R*^2^	∆*R*^2^	∆*F*	*β*
*Low graders from primary school*																	
Step 1	0.46	0.46	10.76***		0.20	0.20	2.46^+^			0.43	0.43	**4.75** **		0.23	0.23	1.83						0.37	0.37	**3.73** *	
age				−0.18				−0.25					−0.21				−0.21								0.03
grade-reason				0.23				0.06					0.04				0.08								0.31
grade-lang				0.10				**0.53** ^*^					**0.54** ^*^				0.45^+^								0.14
RAN			0.52^***^					−0.02					−0.18				−0.18								−0.34
Step 2	0.51	0.05	**2.52** *		0.22	0.02	0.57			0.45	0.02	0.48		0.34	0.11	1.91						0.46	0.09	1.91	
VAS_rt			**−0.27** *					−0.09					−0.13				0.31								0.26
VAS_d′				0.001				−0.11					−0.07				−0.03								0.12
*High graders from primary school*																	
Step 1										0.24	0.24	**4.42** **										0.37	0.37	8.16***	
age													−0.27												−0.09
grade-reason													0.11												−0.03
grade-lang												**0.53** **												**0.61** **	
RAN													−0.04												−0.12
Step 2										0.35	0.11	**4.41** *										0.41	0.04	1.80	
VAS_rt												**−0.35** **													0.02
VAS_d′													−0.04												0.21^+^
*Middle school students*																	
Step 1																	0.10	0.10	1.38						
age																				−0.04					
grade-reason																				0.18					
grade-lang																				−0.01					
RAN																				−0.15					
Step 2																	0.17	0.07	**2.28** ^*^						
VAS_rt																			**−0.30** ^*^						
VAS_d′																				0.16					
*Undergraduates*																	
Step 1								0.04		0.04	0.37						0.16	0.16	**2.48** ^*^						
age												−0.07							−0.32*						
grade-reason												−0.10								0.09					
grade-lang												0.17								−0.09					
RAN												0.12								−0.25^+^					
Step 2								0.18		0.14	**3.19** ^*^						0.28	0.12	**3.98** ^*^						
VAS_rt												−0.36*							**−0.35** **						
VAS_d′												0.20								−0.08					

*Rank-reason, transformed rank data indexing reasoning ability; rank-lang, transformed rank data indexing general language level; RAN, mean time recorded in the rapid naming test; VAS_rt, correct reaction times in the visual 1-back task; VAS_d′, *d*-prime values in the visual 1-back task*.

+ *p < 0.1*;

* *p < 0.05*;

** *p < 0.01*;

*** *p < 0.001*.

*Significant results are in bold*.

To differentiate age-group effects when examining the predictive power of VAS capacity to Chinese reading fluency in the regression models, the differences in the slopes of the relevant regression lines were tested through ANCOVAs following some relevant literature (Huitema, 2011; also, as stated in the Introduction section of Ankarali et al., 2018). We report below the steps of the analysis which proved particularly informative. Given that the VAS skill in both middle school students and undergraduates significantly predicted sentence reading fluency in silent mode, we compared slopes of the two regression lines related to the two age groups. In this ANCOVA, the dependent variable was silent sentence reading speed, the independent variable was a newly established one—age group with two levels in this comparison (i.e., middle school students and undergraduates), and the covariate was VAS skill expressed by correct reaction times in the visual 1-back task. A significant VAS × age group interaction in this ANCOVA would indicate that slopes of the two regression lines for the two age groups are different from each other, which further witnesses a significantly different contribution by VAS skills to silent reading performance between these two groups. Otherwise, if the slopes of the two lines are not different, the inference would be that the ability by VAS capacity to predict reading ability is similar between the two age groups. The results showed that the VAS × age group interaction was not significant [*F*(1, 121) = 0.42, *p* = 0.52], suggesting that there were no significant differences between the slopes of the two regression lines. Similar ANCOVAs were conducted in the following analyses. The comparison of the predictive ability of VAS skills with respect to sentence reading speed in the oral mode between high graders of primary school and adults showed no significant differences, with similar regression lines in terms of slope [non-significant VAS × age group interaction: *F*(1, 134) = 2.01, *p* = 0.14]. Additionally, the VAS skills of adults showed significant prediction for both oral and silent sentence reading speeds. Thus, we compared the slopes of the two regression lines but found no significant difference between them [non-significant VAS × age group interaction: *F*(1, 118) = 2.31, *p* = 0.17].

## Discussion

The present study investigated the developmental changes in the relationship between VAS and Chinese reading fluency through cross-sectional comparisons by recruiting participants ranging from beginning to skilled readers. Cross-grade analyses showed that more advanced readers exhibited faster responses in the VAS test compared to beginning readers (second to sixth grade in primary school). Further position-based analyses showed that all four age groups exhibited the best performance when the target was presented in the center of the string (i.e., position 3), revealing that attentional resources are mostly concentrated in the central area of the string. These findings were consistent with previous studies relying on similar paradigms (Grainger et al., 2016; Schubert et al., 2017; Banfi et al., 2018). Furthermore, the *d*-prime sensitivity in position 3 showed an increasing fixation-position advantage as grade increased. Thus, a typical “reversed V” shaped pattern was observed in the skilled readers. Moreover, the undergraduates also exhibited a left advantage in their attentional distribution, that is, a leftward bias with a significant advantage for the first and second positions over the fourth and fifth positions was observed. Since this leftward bias also tends to increase with age, the developmental increase in the left bias, together with the central fixation advantage, might reflect a change in the shape of visual receptive fields as reading experience increases (Chanceaux and Grainger, 2012) as a result of a complex interplay between crowding effects and a decrease in visual acuity with increasing eccentricity. Indeed, skilled reading involves the parallel processing of characters, often within a single fixation, the location of which is typically slightly left (for left-to-right reading systems) of the center of the string (Rayner, 1979; Ducrot and Pynte, 2002). This preferred and more efficient initial viewing position (the so-called OVP, Optimal Viewing Position, O’Regan and Jacobs, 1992) has been described during the reading of both alphabetic and Chinese scripts (Liu and Li, 2013) and seems to generalize to non-lexical stimuli (nonwords, e.g., Ma et al., 2015, although it is not clear if they also generalize to nonlinguistic symbol strings, see Tydgat and Grainger, 2009). Although the results of some studies using non-verbal symbols (e.g., Tydgat and Grainger, 2009) seem to suggest that this effect is letter specific, other studies suggest that similar effects are found for letter-like (small, high-contrast, and closely spaced, see Spinelli et al., 2002) stimuli. Indeed, the symbols used in the present study do possess such characteristics and are more reminiscent of real Chinese characters than the symbols used by Chanceaux and Grainger, which may be the reason why a leftward bias similar to that found for letter and digit strings is also found with the symbols in the present study. Since the current paradigm used a single-figure target, it might be that the leftward advantage reflects post-stimulus scanning phenomena, as suggested by Aschenbrenner et al. (2017) with letter stimuli (but Ducrot and Pynte, unlike Chanceaux and Grainger, also found such effects for series of hashes). It is also possible that participants developed an expectation to direct their attention to the left side of the visual field prior to the presentation of the string, which might result from their left-to-right orientation during daily reading experience. Although participants were required to keep their gaze on the center of the screen, eye movements were not monitored. However, the performance at the third position of a string was higher than that at the other positions, including the first position. If participants prospectively adjusted their fixation to the leftward figures of a string, then their performance in the first position should be higher than that in the other four positions. Therefore, this possibility may have minimal influence on the leftward-bias pattern in the current cohort of undergraduates. Alternative explanations point to more exquisitely attentional processes: a rapid reallocation of attention toward the beginning of the (word) string as suggested by Aschenbrenner et al. (2017) or an anticipation of a (reading-habit induced) left-to-right visual scanning as suggested by Ducrot and Pynte (2002). Reading experience also appears to be the basis for constantly increasing efficiency at central position: learning to read, in fact, seems to increase the ratio between recognition in the center and recognition away from the center of fixation, as reflected in the changes of the shape of the so-called Form Resolving Field (FRF, which maps the distribution of recognition accuracy onto eccentricity) from symmetrical and reversed-V-shaped to slightly narrower on the side of the reading direction (Zegarra-Moran and Geiger, 1993; Lorusso et al., 2004). This seems very similar to the increase in asymmetry observed in the present study.

We observed that the VAS capacity of the low graders of primary school in the current cohort could significantly predict their single-character reading fluency, while participants from higher grades showed a significant relationship between their VAS skills and sentence reading fluency. That is, the possible role of VAS in the fluent reading procedure may vary with different levels of linguistic structure, indicating a developmental transition in its predictive power from the single-character level to the sentence level. This developmental pattern was consistent with our prediction stated in the last paragraph of the “Introduction” section, referring to the modulation of age-related changes. Based on findings from relevant literature (Bosse and Valdois, 2009) and our current findings, the window size of VAS is approximately 2–3 elements for the lower grades of primary school. Accordingly, it could be speculated that their VAS is narrow and immature, which might correspond to the processing window size of single characters, and thus VAS may play a role in the global visual analysis of Chinese characters by further affecting the reading procedure of single-character processing. Subsequently, VAS broadens with age and is wider for higher graders (Bosse and Valdois, 2009); the larger window size of VAS may allow parallel processing of multiple orthographic units of Chinese characters and affect character position coding and visual-to-semantic mapping, which in turn may have a relation to the efficiency of sentence reading and comprehension at later developmental stages.

In the present study, visual attention span exhibited a significant relation to oral sentence reading fluency in the developing readers and to silent sentence reading speed in the middle school students. As reading experience improves, VAS showed close relations to both silent and oral sentence reading, as observed in the adults. This finding suggests that visual attention span is related to reading fluency in both oral and silent modes; this relationship undergoes a developmental transition starting from the oral reading mode, moving next to the silent reading mode, and finally settling on a relationship to both oral and silent reading modes for skilled readers. Oral reading by primary school students is mainly implicated in the activation of phonological representations and the mapping between orthography and phonology (Jasińska and Petitto, 2014). Because the method of visual script mapping onto speech sounds is addressed in Chinese reading (Tan et al., 2005), in which the visual form of a Chinese character directly and globally corresponds to the syllable, the oral reading procedure might require a narrow VAS window size to further affect the global analysis and decoding of Chinese characters’ visual features. In contrast, silent reading involves simultaneous processing of multiple characters (or of a phrase) to directly map onto the relevant meaning and requires a wider visual attention span. However, the VAS of primary school students is still immature and developing, and so it might not satisfy the requirements of the visual attention window for silent reading, possibly resulting in a non-significant relation between VAS and silent reading. With increasing grade (middle school students), the VAS window size becomes wider, which would contribute to the simultaneous processing of multiple orthographic units. Therefore, VAS might play a role in the visual analysis of multiple characters’ visual forms and, furthermore, in the visual-to-semantic mapping procedure during silent reading. Oral reading in middle school students might still be related to phonological retrieval in character-by-character order (Wang, 2007), which only requires a small visual attention window size; however, the VAS window of middle school students appears to be wider, which does not correspond to the requirements of oral reading, and thus, there is a lower ability of VAS to significantly predict oral fluent reading at this developmental stage. With accumulating reading experience, it has been found that all of the orthographic, phonological, and semantic representations would be activated in the oral as well as the silent reading mode in adults (Jasińska and Petitto, 2014). Neuroimaging findings support the above inference. Relevant results (Wang et al., 2015) show that both silent reading and oral reading in adults activate brain regions involved in visual-to-semantic mapping (e.g., left middle temporal gyrus) and brain areas responsible for orthographic-to-phonological mapping (e.g., left inferior frontal gyrus, left inferior parietal lobule). These findings suggest that silent and oral reading share some cognitive mechanisms. Accordingly, it could be said that the wider size of VAS in the adults might exert an influence on the global processing of visual forms of multiple characters or phrases, further affecting the retrieval of relevant multiple syllables and semantic information through the procedures of visual-to-phonological and visual-to-semantic mapping.

Additionally, the current results provide some evidence for the exploration of the underlying mechanisms of oral and silent reading fluency. In fact, whether the two reading modes involve common or different mechanisms is still a matter of debate. Some researchers suggest that oral and silent reading differ in their cognitive mechanisms. More specifically, oral reading involves more visual-verbal transfer as compared to semantic retrieval, while silent reading depends more on visual-to-semantic decoding than on the orthographic-to-phonological mapping (Galin et al., 1992; Snellings et al., 2009; van den Boer et al., 2014). By contrast, other studies suggest that silent reading and oral reading may essentially involve the same processes, with the exception of additional articulatory demands for oral reading (Ashby et al., 2012). The present findings showed a developmental transition in the role of VAS, from contributing to oral sentence reading, to contributing to silent sentence reading and finally to contributing to both oral and silent sentence reading. Thus, this provides an alternative possibility for the underlying mechanisms of oral and silent reading fluency; that is, their mechanisms might be different from each other at the early developmental stages but may develop to resemble each other with age, and they may both involve visual-to-phonology and visual-to-semantic mapping, in which VAS could play a role.

In summary, cross-grade analyses showed that VAS skills improve with age. In particular, the central fixation advantage and left-side bias became more appreciable with increasing grade, revealing the possible modulation of reading experience. Regression analyses further indicated that VAS is a unique predictor of single-character reading fluency in beginning readers but of sentence reading fluency at later developmental stages. Moreover, VAS could account for the variation of oral reading fluency for developing readers, of silent reading fluency for middle school students, and of both silent and oral reading fluency in adult skilled readers. These developmental transitions might reveal age-related characteristics in the contribution of VAS to reading fluency in languages with deep orthography, such as Chinese. On the whole, the present findings extend our understanding of the role of general perceptual processing (i.e., VAS) in fluent reading from a developmental perspective and provide interesting suggestions about the impact of language transparency on such relationships. It should be noted that we primarily found the reaction times in the VAS task but not the relevant *d*-prime values to exhibit a close relationship with reading fluency. It is possible that the dependent variables of the present study mainly reflect the speed of fluent reading, and thus, the independent variable involving processing speed (i.e., reaction time but not *d*′ values) would exhibit a more significant influence. Additionally, due to the objective time limitation during our data collection, especially for the primary school students, we did not include a single-figure recognition task as a control test. Our previous study did not observe a significant difference in single-element identification between individuals with reading fluency difficulty and age-matched normal readers, and there was no remarkable correlation between the identification of isolated stimuli and reading fluency (Zhao et al., 2018b). These findings suggest the possible absence of a relationship between single-element identification and fluent reading skills. Nevertheless, single-element identification ability should be taken into consideration in future research to ensure that VAS-related measurements reflect performance on simultaneously processing multi-elements rather than identifying single elements. Moreover, the current study used a computerized procedure to measure silent reading speed in the sentence level. Actually, during silent reading, the participants might skip some characters and we could not ensure that they read every character in one sentence to understand its meaning. Hence, the last value that was computed using the ratio of sentence length to reading time might not completely accurately reflect the reading speed in the silent mode. Future studies should take this into consideration and find a more reasonable way to directly measure silent reading speed. Besides, because there were eight measurements in the current correlation analyses, most significant results could not be kept after the Bonferroni correction (the relevant significance would be 0.05/28 ≈ 0.002). Thus, the present conclusions were mainly based on the results of regression analyses instead of the correlation analyses. Furthermore, it should be acknowledged that the current study focused on different age groups by a cross-sectional comparison, and does not shed light on the directionality of the relationship between VAS and fluent reading. Future longitudinal and intervention studies are required to better define the causality of this relationship.

## Data Availability Statement

The datasets generated for this study are available on request to the corresponding author.

## Ethics Statement

The studies involving human participants were reviewed and approved by Research Ethics Committee of School of Psychology, Capital Normal University. Written informed consent to participate in this study was provided by the participants’ legal guardian/next of kin, Capital Normal University.

## Author Contributions

CH collected the data and wrote the manuscript. ML wrote and revised the manuscript. ZL analyzed the data. JZ designed, wrote, and revised the manuscript.

### Conflict of Interest

The authors declare that the research was conducted in the absence of any commercial or financial relationships that could be construed as a potential conflict of interest.
