# Supplementary material for: Developmental Differences in the Relationship Between Visual Attention Span and Chinese Reading Fluency
Source: Front Psychol. 2019 Nov 6;10:2450. doi: 10.3389/fpsyg.2019.02450 (PMC6851167; doi:10.3389/fpsyg.2019.02450)
Supplement: Supplementary file 1 [file Table_1.DOCX]

**Supplementary materials**

In order to check how general reasoning ability affected results of ANOVA on reaction times and d’ values of the visual 1-back task, we further conducted ANCOVAs separately within each age group with position of a string as a within factor and the relevant raw scores of general reasoning ability as covariate. The results were generally similar to the findings without controlling for the covariate of reasoning ability (the results stated in the main manuscript). Detailed information of ANCOVA result was as below.

**Lower graders of primary schools**

After controlling the possible influence of reasoning ability, ANCOVA results on reaction times in the visual 1-back tasks showed that the main effect of position was not significant [*F*(4,332)=2.38, *p*=.13, *η^2^*=.03]. Results on d-prime values showed that the main effect of position was significant [*F*(4,212)=8.49, *p*<.001, *η^2^*=.14]. Post hoc analysis exhibited that the d’ values when targets were presented in the 3^rd^ position were higher than that in the other four positions (*p*s<.05), and there was no any other significant comparisons between positions (*p*s>.1).

**Higher graders of primary schools**

Results of ANCOVA on reaction times showed a non-significant position effect [*F*(4,304)=3.20, *p*=.013, *η^2^*=.04]. Post hoc analysis demonstrated that reaction times in the 5^th^ position were longer than that in the 1^st^ position (*p*=.045) and 3^rd^ position (*p*<.001) without any other significant comparisons (*p*s>.1). Results on d’ values showed that there was a significant main effect of position [*F*(4,236)=12.08, *p*<.001, *η^2^*=.17]. Multiple comparisons exhibited that the d’ values in the 3^rd^ position were higher than that in the other four positions (*p*s<.001) without other significant differences between positions (*p*s>.1).

**Middle school students**

Results on reaction times showed that the main effect of position was significant [*F*(4,248)=4.19, *p*=.003, *η^2^*=.06]. Post hoc analysis reported that the reaction times when the targets appeared in the 3^rd^ position were shorter than that in the 2^nd^, 4^th^, and 5^th^ positions (*p*s<.05) while there was no other significant comparisons between positions (*p*s>.1). Results on d-prime values showed a significant effect of position [*F*(4,208)=16.33, *p*<.001, *η^2^*=.24]. Multiple comparisons exhibited that the d’ values in the 3^rd^ position was higher than that in the other four positions (*p*s<.001) without other significant differences across positions (*p*s>.1).

**Adults**

Results of ANCOVA on reaction times showed a marginally significant effect of position [*F*(4,244)=2.39, *p*=.052, *η^2^*=.04], and post hoc analysis exhibited that reaction times in the 3^rd^ position were shorter than that in 1^st^ position (*p*=.005) and 4^th^ position (*p*=.004) while no other significant comparisons between positions (*p*s>.1). Results on d-prime values showed that the main effect of position was significant [*F*(4,212)=40.77, *p*<.001, *η^2^*=.44]. Multiple comparisons exhibited that the d’ values in the 3^rd^ position were higher than that in the other four positions (ps<.001), and d-prime scores at position 1 and position 2 were higher than that at position 4 and position 5 (ps<.05), without other significant difference across positions (*p*>.1).
